# Chemical Constituents and Structural Characterization of Polysaccharides from Four Typical Bamboo Species Leaves

**DOI:** 10.3390/molecules20034162

**Published:** 2015-03-05

**Authors:** Cheng-Zhang Wang, Hong-Yu Zhang, Wen-Jun Li, Jian-Zhong Ye

**Affiliations:** 1 Institute of Chemical Industry of Forest Products, CAF, Nanjing 210042, China; E-Mails: chemicalzhy@163.com (H.-Y.Z.); liwenjun611@163.com (W.-J.L.); yejianzhong1984@163.com (J.-Z.Y.); 2 National Engineering Laboratory for Biomass Chemical Utilization, Nanjing 210042, China; 3 Key Laboratory of Biomass Energy and Material, Nanjing 210042, China; 4 Institute of New Technology of Forestry, CAF, Beijing 100091, China

**Keywords:** bamboo leaves, polysaccharides, HPLC-GPC, FTIR, NaIO_4_-HIO_4_

## Abstract

In order to find bamboo leaves with high contents of bioactive polysaccharides, 32 samples were chosen to analyze their polysaccharide content by GC and sulfuric acid-anthrone colorimetric assays. Purified polysaccharides (BLPS) were separated from the four varieties *P. nigra* (Lodd.) Munro (PN), *P. vivax* McClure (PV), *Chimonobambusa quadrangularis* (Fenzi) Makino (CQ), and *P. bambussoides* cv. Tanakae (PB) by ultrasound extraction, solution precipitation, ion exchange resin, DEAE-52 and Sephadex G-100 chromatography. BLPS structural characterization was accomplished by HPLC-GPC, Fourier transform infra-red spectroscopy (FTIR) and NaIO_4_-HIO_4_ oxidation reactions. The results showed that the total polysaccharides of the bamboo leaves in samples 1–32 ranged between 1.4% and 5.4%, Samples No. 29–No. 32 (PN, PV, CQ, and PB) contained 2–3 fold more polysaccharides than No. 1~No. 28 among the 32 different species, particularly the content of galactose was in a range of 21.5%–34.1% for these four typical bamboo species leaves, which was also more than 2–3 fold higher than in No. 1–No. 28. Sugar analysis indicated that PN-PBLPS-1, PV-PBLPS-1, CQ-PBLPS-1 and PB-PBLPS-1 from the four varieties were homogeneous polysaccharides with molecular weights of 2.04 × 10^4^, 1.15 × 10^4^, 8.75 × 10^4^ and 1.48 × 10^4^ Da, respectively. PB-PBLPS-1 was a mixture of α-galactopyranose and β-d-glucopyranose linkages with α-(1→6) or β-(1→6)glycosidic bonds, while PN-PBLPS-1, PV-PBLPS-1, and CQ-PBLPS-1 had α galactopyranose linkages with α-(1→6) glycosidic bonds.

## 1. Introduction

Bamboo belongs to the Gramineae Bambusoideae subfamily and is one of the most valuable evergreen perennial plants in China and South-east Asia. China has been known as the “bamboo kingdom”, and bamboo timber is a traditional forest product containing high cellulose, hemicellulose and lignin used as a construction material [1].

Generally hemicelluoses are a family of polysaccharides which contain a backbone of d-xylopyranosyl residues, linked together by β-(1→4)-glycosidic bonds [2], while the bioactive polysaccharides in bamboo are oligosaccharides of β-d-glucan and xyloglucan or *p*-coumaroyl arabinoxylan [3]. The investigation showed that bamboo shoot polysaccharides are composed of arabinoxylan (1→3,1→4)-β-d-glucan, xyloglucan and glucomannan [4], and other kinds of arabino-glucuronoxylans are also isolated from bamboo shoot with a linear (1→4)-β-d-xylopranosyl backbone to which α-l-arabinofuranose units or short chains of 4-*O*-methylglucuronic acid. However, these hemicellucosic polysaccharides have a average molecular weight (M_r_) range of 10 × 10^4^–40 × 10^4^ Da, and this results in very weak physiological activity.

In order to get strongly bioactive polysaccharides with a average M_r_ range of less than 10 × 10^4^ Da, scientists often use chemical or biological ways to degradate macromolecular polysaccharides. This causes serious environmental pollution and results in high costs, therefore, it is very important to seek highly bioactive polysaccharides with low molecular weight from forestry resources or biomass residues. Apart from bioactive compounds like flavonoids, glycosides and polyphenols, bamboo leaves contain a large amount of active polysaccharides [5,6,7]. Extract of bamboo leaves (EBL) has been proved very safe and is used widely in China as an antioxidant in food additives [8]. The key bioactive compounds of EBL are polysaccharides and flavonoids, where particularly bamboo oligosaccharides play important functions [9,10].

The polysaccharide of bamboo leaves (BLPS) is a kind of bioactive heteropolysaccharide with medium molecular mass. BLPS was first isolated from Japanese bamboo leaves of *Sasa kurilensis* and *Sasamorpha chiisanensis* [11]. The reported average molecular mass M_r_ of the BLPS was determined to be in a range of 2 × 10^4^–3 × 10^4^ Da, and this BLPS was proven to have good antitumor activity. Kato separated β-d-glucan and xyloglucan bioactive polysaccharides from bamboo shoots [12], while Suzuki also obtained from green bamboo shoots a bioactive hemicellulose polysaccharide with strong inhibition on S_180_ tumors [13]. Tang extracted and purified polysaccharides with an average molecular weight (M_r_) range of 10^3^–10^4^ Da from bamboo leaves of *Phyllostachys heterocycla* cv. pubescens, and it had very strong physiological activity [9]. The bioactive BLPS is composed of xylose, arabinose, glucose and galactose, as well as lesser amounts of mannose, rhamnose and uronic acids. In recent years, Ye investigated the total content and chemical constituents of polysaccharides from 17 kind species of bamboo leaves from the Nanjing region, Jiangsu Province. The total content of polysaccharides was between 1.9%–5.7% [14,15]. Lu found polysaccharide levels approaching 14.55% in bamboo leaves of *Sinocalamus latiflorus* McClure [16].

The composition and content of BLPS show very large changes for different varieties, growth place, harvest season, and growth ages. However, so far, there has been little investigation concerning the chemical composition and structural characterization of BLPS from different varieties in China with mono-sugar links, and their average M_r_ and bioactivity.

In this paper, 32 varieties of bamboo leaves from Zhejiang Province in South-East China were chosen to investigate the content of polysaccharides obtained by ultrasound-assisted extraction. *Phyllostachys nigra* (PN), *Phyllostachys vivax *(PV), *Chimonobambusa quadrangularis (Fenzi) Makino* (CQ) and *Phyllostachys bambusoides* (PB) have been screened to investigate the structural characterization of the purified bamboo leaves polysaccharides (PBLPS) obtained after solution precipitation to remove proteins, ion exchange resin bleaching, and finally purification using DEAE-52 and Sephadex G-100 chromatography. This investigation is of significance to develop new healthcare foods or medicines from BLPS by using the processed bamboo residues.

## 2. Results and Discussion

### 2.1. The Components and Content of Polysaccharides in Different Bamboo Leave Varieties 

**Figure 1:**
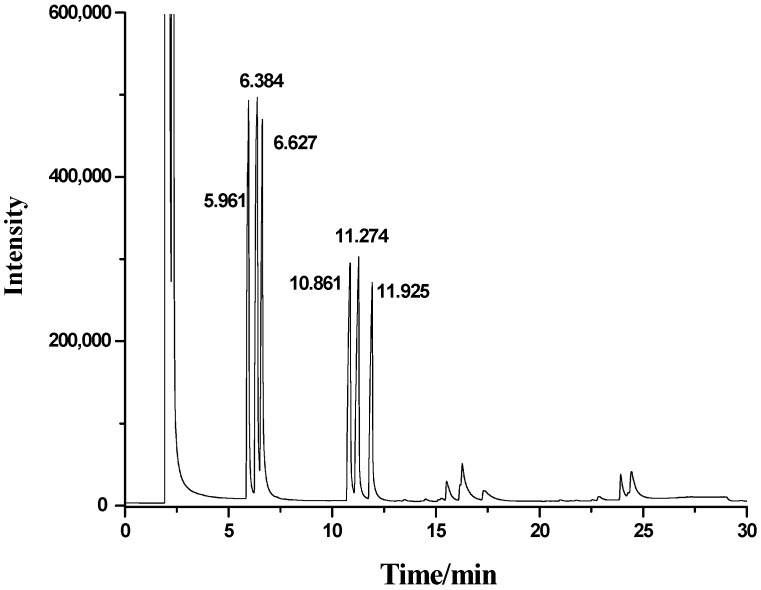
GC trace of standard monosaccharides.

Six kinds of standard monosaccharides were mixed, derivatized and checked by gas chromatography as seen in Figure 1.

The retention times of the monsaccharides were determined as 5.961 min for rhamnose, 6.384 min for arabinose, 6.627 min for xylose, 10.861 min for mannose, 11.274 min for glucose and 11.925 min for galactose (Figure 1). A standard curve of glucose was obtained from a linear regression of the content of glucose (C, mg) and absorbance value (A) (Equation (1)).

A = 5.8137C − 0.00473(R^2^ = 0.9987) (1)

The regression was tested and showed a good linear relation in the content range of 0.0–96.0 μg/mL glucose. By comparison with the relative retention times of the six monosaccharide standards, the monosaccharide composition and their relative contents in 32 species of bamboo leaves were then determined by GC. The BLPS of samples No. 1–No. 32 is mainly composed of 42.6%–62.7% of xylose, 10.2%–24.6% of arabinose, 10.2%–21.2% of glucose, 7.7%–15.7% of galactose and very little rhamnose and mannose. Mannose was not detected in 11 samples: Nos. 3, 4, 7, 8, 10, 11, 13, 14, 18, 19, and No. 26, rhamnose and mannose both were not found in 10 samples: Nos. 15–17, 20–22, and 29–32, while other samples just had less than 6.7% of rhamnose and less than 2.2% of mannose. Another discovery was that samples No. 29–No. 32 of *P. nigra* (Lodd.) Munro (PN), *P. vivax* McClure (PV), *Chimonobambusa quadrangularis* (Fenzi) Makino (CQ), and *P. bambussoides* cv. Tanakae (PB) not only consisted of four kinds of monosaccharides (xylose, arabinose, glucose and galactose), but the content of galactose is also in a range of 21.5%–34.1%, which is 2–3 fold more than in samples No. 1–No. 28. Beside the chemical composition, a sulfuric acid-anthrone colorimetric assay showed the total polysaccharides content of the 32 samples was between 1.4%–5.4%, and the total polysaccharides of PN, PV, CQ and PB ranged between 4.2%–5.4%, meaning that among the 32 different species listed in Table 1 samples No. 29–No. 32 contained 2–3 fold more polysaccharides than No. 1–No. 28. Therefore, these four typical species were chosen to investigate the structural characterization of BLPS.

**Table 1:** The content of total polysaccharides and monosaccharides from 32 different varieties of bamboo leaves.

No.	Varieties of Bamboo Leaves	Total Polysaccharides (%)	Rhamnose (%)	Arabinose (%)	Xylose (%)	Mannose (%)	Glucose (%)	Galactose (%)
1	*P. praecox* C.D. Chu et C.S. Chao	1.4	3.2	14.0	48.8	4.7	15.9	13.1
2	CV. Ventricousinternode	1.5	4.6	16.3	50.3	1.4	17.2	10.1
3	*Bambusa multiplex* cv. Alphonse-Karr	1.5	2.7	16.4	44.2	-	20.7	15.7
4	*P. elegans* McClure	1.6	1.1	15.9	56.1	-	15.3	11.3
5	*Sasa pygmaea* (Miq.) E. G. Camus	1.6	2.5	17.4	55.5	0.9	13.6	9.7
6	*Pseudosasa viridula* S. L. Chen et G. Y. Sheng	1.9	6.7	13.8	50.2	1.6	18.4	10.6
7	*P. heterocycla* (Carr.) Mitford cv. Pubescens	1.9	2.6	14.2	58.5	-	16.1	8.3
8	*P. vivax* McClure Aureocaulis	1.9	1.2	14.5	59.2	-	14.7	10.2
9	*P. viridis*	2.0	2.1	15.2	62.7	0.8	10.2	8.6
10	*Bambusa albo-lineata* (McClure) Chia	2.2	1.8	20.5	56.0	-	12.3	9.1
11	*P. propinqua* McClure	2.2	1.1	17.0	56.2	-	15.1	10.2
12	*P. glauca* McClure	2.2	1.4	14.5	57.5	1.3	16.0	8.9
13	*Phyllostachys*	2.2	2.2	18.0	54.6	-	16.8	7.7
14	*P. heterocycla* cv. Huamozhu	2.3	0.6	14.2	56.4	-	19.9	8.5
15	*P. propinqua* McClure	2.5	-	14.7	56.7	-	17.6	10.8
16	*Bambusa ventricosa* McClure	2.7	-	19.6	52.8	-	16.2	11.4
17	*Bambusa multiplex* (Lour.) Raeusch. ex Schult.	2.8	-	17.6	48.9	-	20.6	12.9
18	*Sinobambusa* tootsik(sieb.) Makino	2.8	2.3	15.9	47.6	1.6	20.0	12.4
19	*P. heterocycla* (Carr.) Mit Ford	2.8	1.3	13.6	58.0	-	16.4	10.4
20	*Pseudosasa amabilis* (McClure)	2.9	-	18.6	60.2	-	11.7	9.5
21	*P. aureosuleata* McClure cv. Pekinensis	2.9	-	14.5	57.5	-	19.0	8.9
22	*P. iridescins* C.Y.Yao et S.Y.Chen	2.9	-	17.2	57.2	-	16.8	8.5
23	*P. aureosulcata* f. spectabilis	3.1	6.7	10.2	48.9	1.2	21.2	11.5
24	*P. gramineus* (Bean) Nakai	3.2	1.8	15.7	53.7	0.6	19.6	8.2
25	*Bambusa rutila* McClure	3.3	3.7	11.3	55.9	0.4	18.1	10.5
26	*Pleioblastus amarus* (Keng) keng	3.7	0.8	21.2	52.4	-	14.2	11.4
27	*Pleioblastus kongosanensis* f. aureostriaus	3.9	4.7	17.6	47.9	0.5	16.2	12.9
28	*Cyperus alternifolius*	3.9	5.3	15.7	53.7	2.2	12.3	10.8
29	*P. nigra* (Lodd.) Munro	5.4	-	15.4	30.2	-	20.1	34.1
30	*P. vivax* McClure	4.7	-	21.3	33.2	-	17.4	27.8
31	*Chimonobambusa quadrangularis* (Fenzi) Makino	4.6	-	21.6	29.8	-	16.8	31.4
32	*P. bambussoides* cv.Tanakae	4.2	-	24.6	32.6	-	20.8	21.5

### 2.2. Purification of BLPS by Sevag and Ion Exchange Resin

The pulverized samples of the four varieties of bamboo leaves were extracted in hot water with the aid of ultrasound. The yield of polysaccharide extract was over 98%. Decolorized polysaccharides were prepared from the precipitate fraction (DPS-1) and the clear filtrate (DPS-2) by ethanol precipitation, centrifugation and resin decolorization. The Sevag method (chloroform-*n*-butanol = 4:1, v/v) could remove over 95% of the free bamboo protein in 80% ethanol. In general, the decolorization of plant polysaccharides is very difficult to accomplish due to the presence of mixed deep colored plant polyphenols. The typical decolorization methods used are ion exchange, oxidation, metal complexes, and adsorption (cellulose, diatomaceous earth, activated carbon, *etc.*).

**Figure 2:**
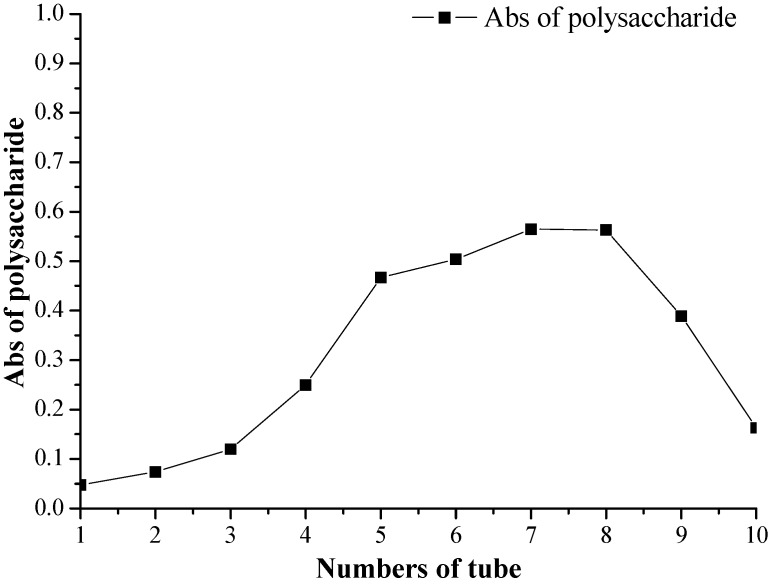
The dynamic adsorption of polysaccharide solution of *Phyllostachys nigra* (PN*)* on D941.

Ion exchange resins are the most commonly used decolorizing agents, and can adsorb yellow pigments like flavonoids, carotenoids, cyanidins *etc.* and plant polyphenols. Compared with the weakly polar macroporous resin D860021 and non-polar macroporous resin DM-2, the macroporous anion exchange resin D941 has the best decolorization effect on crude polysaccharide extracts and gave the highest decolorization rate of 70.30%. The retention rate of polysaccharides approached 86.37%, while the decolorization rates achieved with D860021 and DM-2 were just 42.85% and 19.93%, respectively. The key reason was that the colored impurities are mostly positively changed polyphenol molecules, which are very easily absorbed and exchanged to form ionic bonds with the weakly basic anionic resin. Therefore, the yields of DPS-1 and DPS-2 were with on average 3.2%–3.8%. The dynamic adsorption curve of D941 is shown in Figure 2. When a crude polysaccharide solution of 25 mg/mL was passed through the D941 column at a speed of 3–5 mL/min, and the effluent volume of the sample approached 2.55 times the column volume, the resin reached saturation, this was the point was where the D941 column absorbed the polysaccharides.

### 2.3. Seperation of DPS by DEAE-52 Cellulose and Sephadex Gel Chromatography

**Figure 3:**
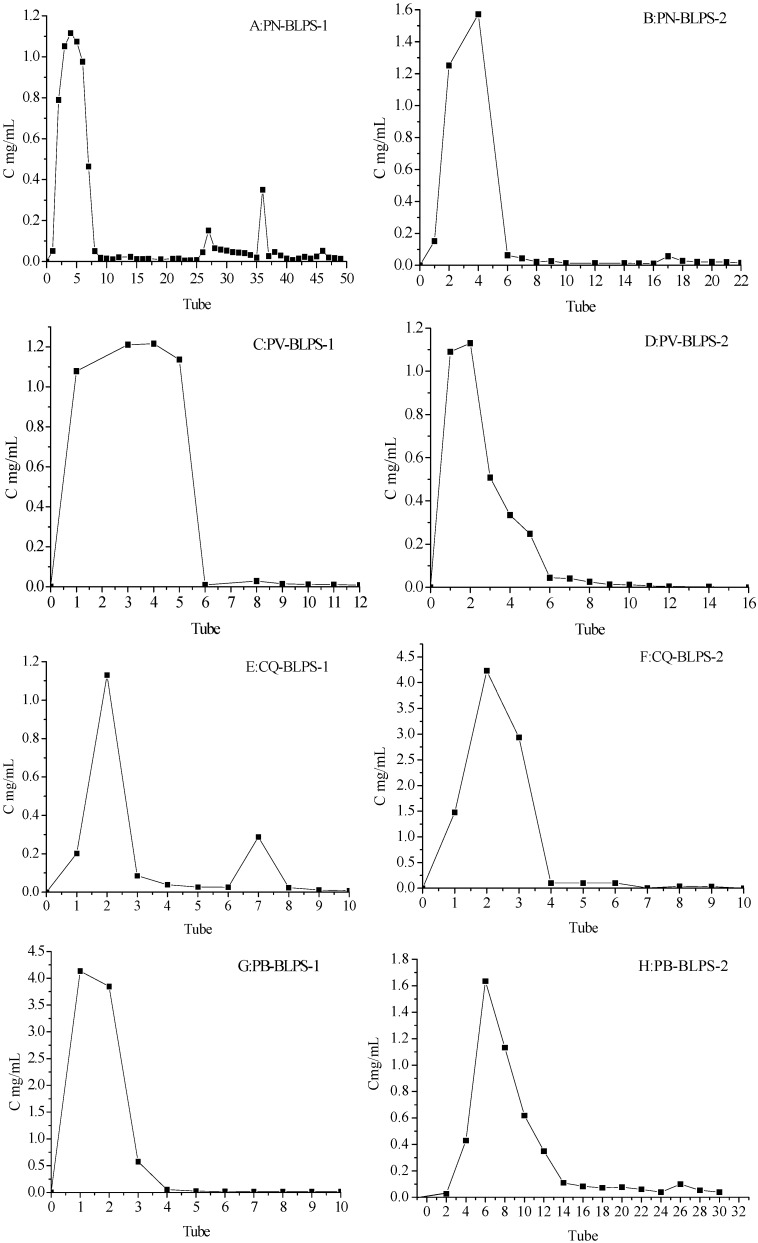
Separation of DPS from four varieties of bamboo leaves. (**A,B**) from *Phyllostachys nigra* (PN); (**C,D**) from *Phyllostachys vivax* (PV); (**E,F**) from *Chimonobambusa quadrangularis* (Fenzi) Makino (CQ), (**G,H**) from *Phyllostachys bambusoides* (PB) by a DEAE-52 cellulose column.

A DEAE-52 cellulose column was adopted to separate DPS from the four varieties PN, PV, CQ and PB. The DEAE-52 column was eluted with a gradient of aqueous NaCl solution (0.1, 0.3 and 0.5 mg/mL) at a flow rate of 1 mL/min. Due to the low yields and deep color of the NaCl eluate, the emphasis was to investigate the water eluate, and the largest peak was collected from tubes No. 1–No. 14 to separate the highest concentration of polysaccharides (Figure 3). The total polysaccharides of BLPS-1 and BLPS-2 ranged from 65.55%–72.83% and 70.23%–78.77% by DEAE-52 cellulose separation of DPS-1 and DPS-2, respectively.

**Figure 4:**
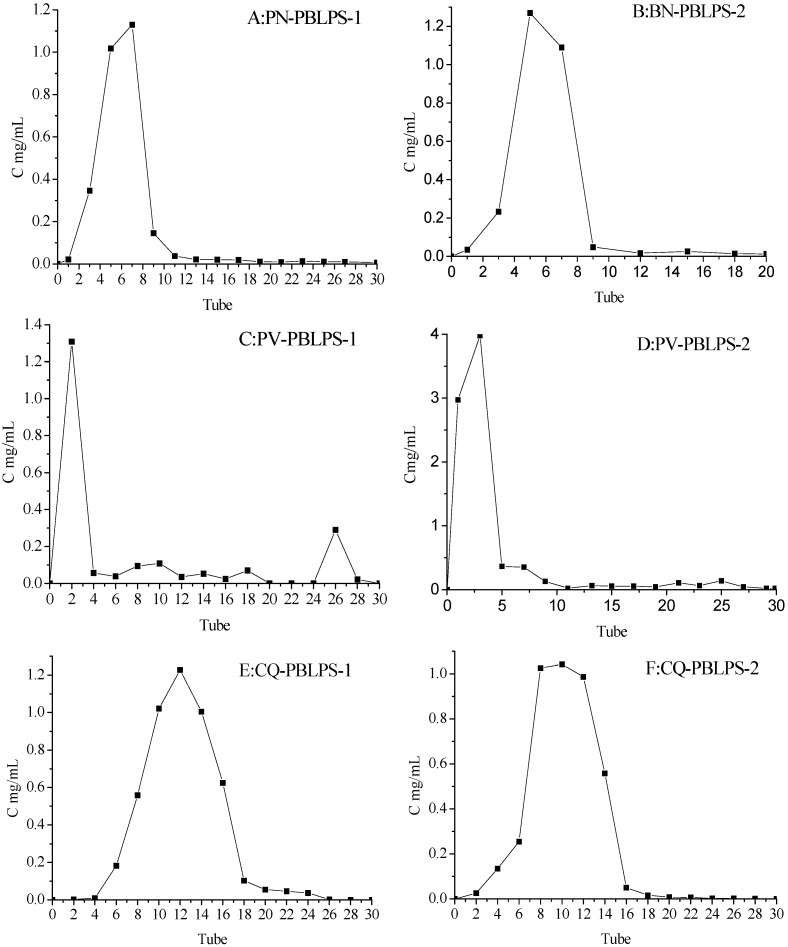

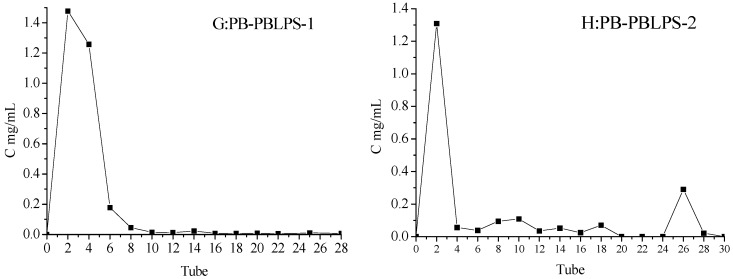
Enrichment and separation of BLPS by Sephadex G-100 from the four kind viarieties of bamboo leave: PBLPS: Purified BLPS. (**A,B**) from *Phyllostachys nigra* (PN); (**C,D**) from *Phyllostachys vivax* (PV); (**E,F**) from *Chimonobambusa quadrangularis (Fenzi) Makino* (CQ); (**G,H**) from *Phyllostachys bambusoides* (PB).

In order to obtain high purity polysaccharide, the water-eluted polysaccharide fractions of the DEAE-52 cellulose column were further separated by Sephadex G-100 washed with H_2_O and NaCl solution. Most of the BLPS was concentrated in the water eluate, and only a small amount of polysaccharide was distributed in the NaCl elute (PV and PB), so the largest peak was collected from tubes No. 1–No. 10 to obtain 90.36%–91.28% of polysaccharides from PN, tubes No. 1–No. 6 to obtain 94.51%–95.33% of polysaccharides from PV, tubes No. 4–No. 18 to obtain 92.77%–93.91% of polysaccharides from CQ and tubes No. 1–No. 6 to obtain 96.20%–96.87% of polysaccharides for PB in Figure 4. Then the high purity PBLPS-1 and PBLPS-2 separated by Sephadex G-100 was used for the identification of their chemical structure by gel chromatography, Fourier transform infra-red spectroscopy (FTIR) and NaIO_4_-HIO_4_ oxidation reactions.

### 2.4. Molecular Weight of PBLPS

Glucose dextran-T standards show a very good linear relationship between the logarithm of the standard molecular weight in a range of 180–160 × 10^4^ and retention time. The standard calibration curve for the series of dextran-T compounds gave a regression coefficient, *R*^2^ = 0.9990 (Equation (2)):

y =−0.4644x + 9.6391 (*R*^2^ = 0.9990) (2)

**Figure 5:**
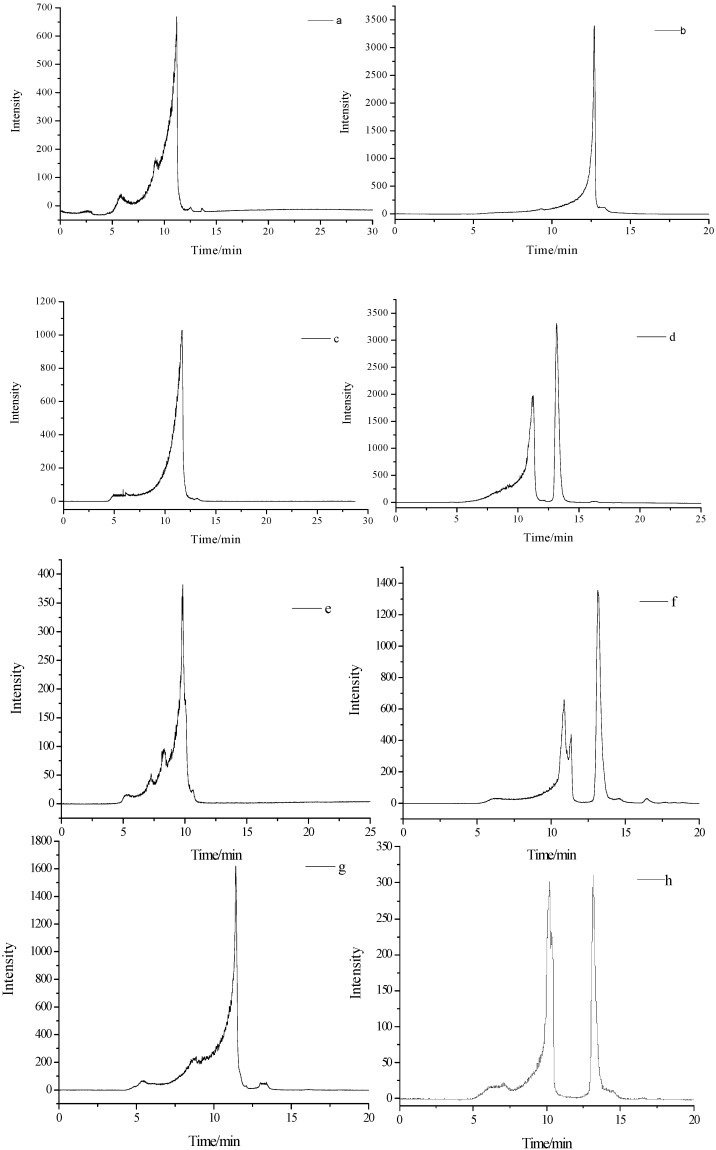
GP-HPLC of PBLPS from the four varieties of bamboo leave: PBLPS: Purified BLPS. (**a,b**) from *Phyllostachys nigra* (PN); (**c,d**) from *Phyllostachys vivax* (PV); (**e,f**) from *Chimonobambusa quadrangularis* (Fenzi) Makino (CQ); (**g,h**) from *Phyllostachys bambusoides* (PB).

The molecular weight of PBLPS-1 and PBLPS-2 from the four varieties of bamboo leaves was determined by using GPC HPLC (Figure 5). This showed that PN-PBLPS-1, PN-PBLPS-2, PV-PBLPS-1, CQ-PBLPS-1 and PB-PBLPS-1 were homogeneous polysaccharides with molecular weights of 2.04 × 10^4^, 3.51 × 10^3^, 1.15 × 10^4^, 8.75 × 10^4^, and 1.48 × 10^4^, respectively, while the others were heterogeneous polysaccharides. PV-PBSP-2 contained two kinds of polysaccharides with molecular weights of 1.89 × 10^4^ and 2.06 × 10^3^, CQ-PBSP-2 and PB-PBSP-2 consisted of three types of polysaccharide. CQ-PBSP-2 was a mixture of components with molecular weights of 2.72 × 10^4^, 1.60 × 10^4^ and 2.07 × 10^3^. PB-PBSP-2 also had three compounds with molecular weights of 5.85 × 10^4^, 4.88 × 10^4^ and 2.07 × 10^3^. The results also showed that molecular weight of PBSP-1 obtained from the alcohol precipitation was higher than that of PBSP-2 from the alcohol filtrate. The key reason for this is the different separation methods used. High molecular weight PBSP-1 was a homogeneous polysaccharide, while small molecular weight PBSP-2 was a heterogeneous polysaccharide. Due to more complex constituents and structure of PBSP-2, further studies on the structure and bioactivity of PBSP-2 will be performed in the future.

### 2.5. Fourier Transform Infra-Red (FTIR) Spectroscopy of PBLPS

**Figure 6:**
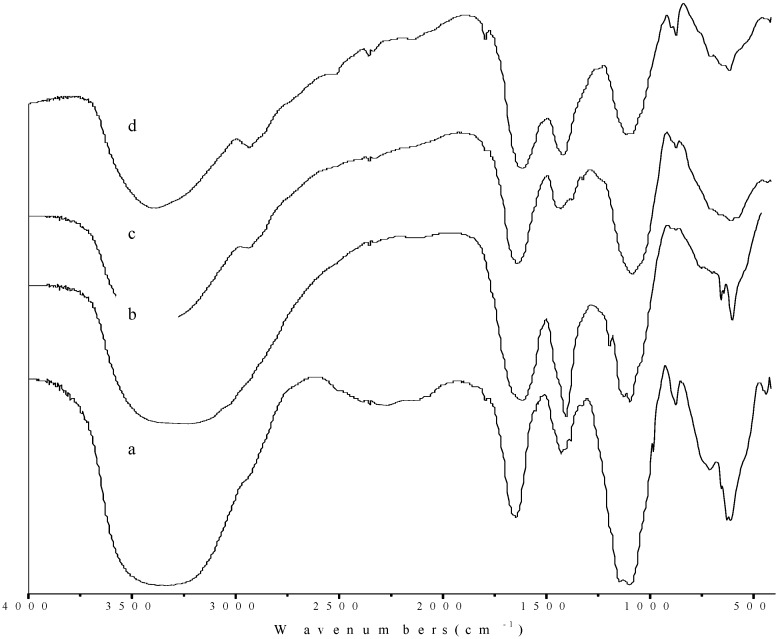
FTIR of PBLPS-1 from the four varieties of bamboo leave: (a) PBLPS-1 of *Phyllostachys nigra* (PN); (b) PBLPS-1 of *Phyllostachys vivax* (PV); (c) PBLPS-1 of *Chimonobambusa quadrangularis* (Fenzi) Makino (CQ); (d) PBLPS-1 of *Phyllostachys bambusoides* (PB).

As PBSP-1 was a homogeneous polysaccharide, it was further investigated to elucidate its structure elucidation. Figure 6 shows the FTIR spectra of PBLPS from the four kinds of bamboo leaves. It was found that the four kinds of PBSP-1 had similar IR absorption bands as follows (cm^−1^): 3375.37, 1647.53, 1430.02, 1385.56, 1099.54 and 855.08 for PN-PBLPS-1; 3230.70, 1617.45, 1405.52, 1126.08, 1096.84 and 856.05 for PV-PBLPS-1; 3395.95, 2937.59, 1790.30, 1643.66, 1432.21, 1377.1, 1084.46 and 853.55 for CQ-PBLPS-1; 3387.91, 2932.82, 1795.70, 1614.00, 1419.18, 1115.99, 897.09 and 853.82 for PB-PBLPS-1. Broad peaks in the 3230.70–3395.95 cm^−1^ range are assigned to the hydroxyl stretching vibration. The absorption at 2937.59–2937.59 cm^−1^ is assigned to C-H stretching vibration, the shoulder peak at 1790.30–1795.70 cm^−1^ is due to the C=O stretching vibration in polysaccharide polymers, while the peak at 1614.00–1647.53 cm^−1^ is also a C=O stretching vibration of a -CHO group, which is a characteristic absorption peak of uronic acid structures. The absorption of 1405.52–1432.21 cm^−1^ is the C-O stretching vibration of a –COOH group, the peak at 1377.10–1385.56 cm^−1^ is the C-H deformation vibration. Absorptions at 1115.99~1126.08 cm^−1^ correspond to the bending vibration of the C-OH group and the peak at 1084.46–1099.54 cm^−1^ is the C-O-C stretching vibration, which are characteristic absorption peaks of pyranoside linkage structures [17,18].

In carbohydrate analysis by IR, α and β conformers can be clearly distinguished by the anomeric region-vibrational bands in the 950 to 750 cm^−1^ region, where 870–840 cm^−1^ corresponds to the α configuration, while the β configuration lies around 890 cm^−1^ [19,20]. In this study, the peak at 853.55–856.05 cm^−1^ is the C-H bending vibration of α-galactopyranose linkages in all PBLPS, while the 1.897.09 cm^−1^ and 710.74 cm^−1^ absorption peaks are the C-H deformation vibration and C-O-C stretching vibrations of β-d-galactopyranose linkages only present in PB-PBLPS-1, so apart from PB-PBLPS-1, which is a mixture of α-galactopyranose and β-d-glucopyranose linkages, the others (PN-PBLPS-1, PV-PBLPS-1, CQ-PBLPS-1) just have α-galactopyranose linkages. All PBLPS-1 are homogeneous polysaccharides with galactopyranose linkages. According to the molecular weight of PBLPS-1, the polymerization numbers of galactose units are 113, 64, 486, 82 for PN-PBLPS-1, PV-PBLPS-1, CQ-PBLPS-1, and PB-PBLPS-1, respectively.

### 2.6. NaIO_4_-HIO_4_ Oxidation and Smith Degradation of PBLPS-1

NaIO_4_-HIO_4_ oxidation was used to determine the glycosidic bond linkages and non-carbohydrate residues in polysaccharides by estimating the number of moles of periodate consumed and the amount of formate produced in the reaction. Periodate oxidation involves the simultaneous oxidation and cleavage of carbon to carbon bonds that have adjacent free hydroxyl groups in polysaccharides, the products of periodate oxidation include homologous aldehydes of the polysaccharide, formaldehyde or formate, The cleavage reaction of one molecule of carbon to carbon bonds consumes one molecule of periodate. The NaIO_4_-HIO_4_ standard curve showed a very good linear relationship between the standard concentration and UV absorbance at 223 nm. The standard calibration curve for the consumption of NaIO_4_ gave a regression coefficient, *R*^2^ = 0.9992 (Equation (3)):

y = 0.0327x − 0.0077(*R*^2^ = 0.9992) (3)

**Figure 7:**
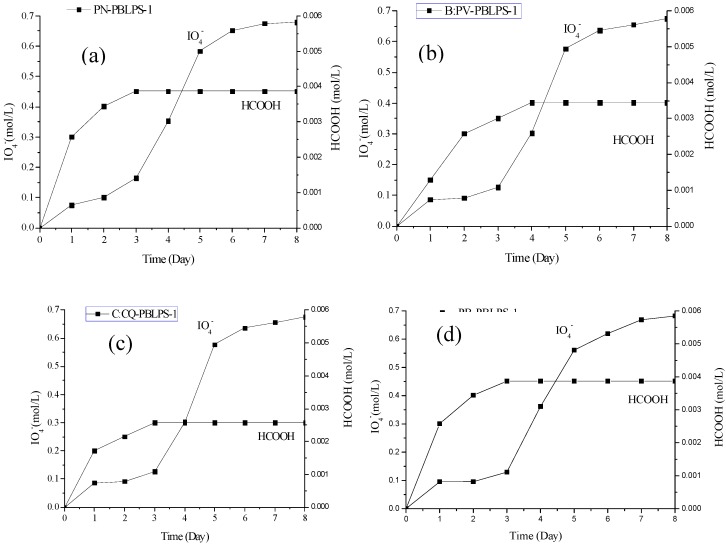
Consumption of IO_4_^−^ and production of HCOO for PBLPS-1 from the four varieties of bamboo leave: (**a**) PN-PBLPS-1 of *Phyllostachys nigra* (PN) (**b**) PV-PBLPS-1 of *Phyllostachys vivax* (PV) (**c**) CQ-PBLPS-1 of *Chimonobambusa quadrangularis (Fenzi) Makino* (CQ) (**d**) PB-PBLPS-1 of *Phyllostachys bambusoides* (PB).

The consumption of IO_4_^−^ and production of HCOO for PBLPS-1 from the four kinds of bamboo leaves is shown in Figure 7. The amount of formic acid generated became almost stable 3 days later, and IO_4_^−^ consumption was basically unchanged after 6 days. All PBLPS-1 were oxidized into formic acid. IO_4_^−^ consumption was twice as great as the amount of formic acid, which indicated that all PBLPS-1 mainly contained (1→6)galactopyranose linkages. All PBLPS-1 were shown to be homogeneous polysaccharides with galactopyranose linkages and an average molecular weight (M_r_) range of 1.15 × 10^4^−8.75 × 10^4^, while all PBLPS-2 were heterogeneous polysaccharides with a small M_r_ range of 2.06 × 10^3^−5.85 × 10^4^. 

## 3. Experimental

### 3.1. Materials

Thirty two samples of bamboo leaves were collected from Anji Country in Zhejiang province of Southern China in October, 2012. They were dried in an oven under circulated air at 50 °C for 24 h before use and crushed into powder of 60–80 mesh particle size.

### 3.2. Experimental Apparatus and Chemicals

Sulfuric acid-anthrone (anthrone 0.2 g and 80% sulfuric acid 100 mL) was used to determine the content of polysaccharides in bamboo leaves using a 722 spectrophotometer (Shanghai Spectrum Instruments Co., Ltd, Shanghai, China). The composition and structure of BLPS were elucidated by GC using a Shimadzu (Shimadzu (China) Co., Ltd., Beijing, China) instrument. Fourier Transform Infra-Red (FTIR) and Gel HPLC were from Waters (Milford, MA, USA). The standard compounds of rhamnose, arabinose, xylose, mannose, galactose, glucose were purchased from Sigma Chemical Company (Beijing, China) and other chromatographic reagents were purchased from local chemical suppliers.

### 3.3. Experimental Methods

#### 3.3.1. Colorimetric Analysis of BLPS

Different samples of bamboo leaves (2.0 g) were weighed into a round bottom flask, and extracted with the aid of ultrasound at a solid-liquid ratio of 1:10 (g/mL), two times, 30 min at 80 °C each time. The extracting solution was mixed and condensed under vacuum, then diluted with distilled water to 25 mL in a volumetric flask, then 1.0 mL of the diluted solution was placed in a 10 mL tube with stopper. Then, the steps above were repeated. The polysaccharide content of bamboo leaves (BLPSC) were calculated using Equation (4). Anthrone (0.2 g) was diluted with 80% sulfuric acid to 100 mL and mixed, then a quantified amount of glucose was diluted to 0.12 mg/mL with distilled water. The standard solution of glucose was divided into portions of 0.0, 0.1, 0.2, 0.3, 0.4, 0.6 and 0.8 mL in 10 mL tubes with stopper, and then diluted in turn to 10 mL with deionized water. Then, 4 mL sulfuric acid-anthrone was measured out and added to each tube cooled with ice, reacted for about 10 min in boiling water, then cooled to ambient temperature. The absorbance value (A) was measured at 620 nm:

(4) $$\text{BLPSC}\;=\;\frac{C\times v}{w}\times 100\%$$

C: concentration of polysaccharides from the linear regression equation, μg/mL
V: volume of polysaccharide, mL
w: sample weight of bamboo leaves, g

#### 3.3.2. Gas Chromatography

The samples were hydrolyzed with 1.5% H_2_SO_4_ solution, and derivatized by acetylation according to [9]. A GC system (Shimadzu (China) Co., Ltd.) instrument) was used to analyze the monosaccharide composition of the bamboo sample polysaccharides using a HP-5 capillary column (30 m × 0.32 mm × 0.25 μm) at 250 °C of oven temperature and 300 °C of detector temperature with FID detector and 1 mL/min of carrier gas. Temperature programming: 180 °C→2 °C/min→220 °C (1 min)→5 °C/min→250 °C (2 min).

#### 3.3.3. Extraction and Purification of BLPS

Pulverized samples (200 g) of the four varieties of bamboo leaves: *Phyllostachys nigra* (PN), *Phyllostachys vivax* (PV), *Chimonobambusa quandrangularis (Fenzi) Makino* (CQ) and *Phyllostachys bambusoides* (PB), were extracted with the help of ultrasoound wave with water at a solid-liquid ratio of 1:10 (g/mL), two times, 30 min at 80 °C each time. The extracting solutions were mixed and concentrated under vacuum. 80% ethanol was added to the concentrated solution with continuous stirring, and the precipitated fractions were centrifuged to produce the crude polysaccharides extract, then the crude extract was washed with hydrochloric acid to remove proteins, and decolorized with D941 resin and lyophilized, to give the extract of decolorized polysaccharides (DPS-1) according to [11]. The same purification method was used to get the clear filtrate from 80% ethanol precipitated crude polysaccharide. The clear filtrate was condensed, then 80% ethanol was added to precipitate the polysaccharides of the concentrate and centrifuged again. The extract of decolorized polysaccharides (DPS-2) was also prepared from the clear 80% ethanol filtrate.

#### 3.3.4. Ion-Exchange and Sephadex Gel Chromatography of DPS

DPS-1 and DPS-2 from the four kinds of bamboo leaves, respectively, were dissolved in deionized water with a polysaccharide concentration of 15 mg/mL, the solution was loaded onto the treated DEAE-52 cellulose column (3.8 cm i.d. ×120 cm, Pharmacia, Shanghai, China) equilibrated with deionized water. Then the DEAE-52 column was eluted with a gradient of aqueous NaCl solution (0.1, 0.3, and 0.5 mg/mL) at a flow of 1 mL/min. Eluted fractions were collected in 20 mL tubes and the polysaccharide content was monitored by the anthrone-sulfuric acid method.

The polysaccharide fractions eluted from the DEAE-52 cellulose were lyophilized, then dissolved in deionized water with a polysaccharide concentration of 5 mg/mL, and the solution was loaded onto a treated Sephadex G-100 coumn (2.2 cm i.d. ×80 cm, Pharmacia), then washed with H_2_O at a flow rate of 0.33 mL/min. The same method was used to analyze the polysaccharide content of the eluted fractions collected in 20 mL tubes. The purified polysaccharide fractions (PBLPS) were concentrated and lyophilized, then PBLPS-1 and PBLPS-2 were prepared for the identification of their chemical structure by gel chromatography, Fourier transform infra-red spectroscopy (FTIR) and oxidation with NaIO_4_–HIO_4_ [20].

#### 3.3.5. Determination of Relative Molecular Weight of PBLPS

Every sample of PBLPS-1 and PBLPS-2 (3.0 mg) was dissolved in of distilled water (2.0 mL) at 80 °C by the aid of ultrasound for 5 min, then filtered through a 0.45 μm membrane. The molecular weight of PBLPS-1 and PBLPS-2 was determined using gel permeation HPLC equipment (Agilent 1200) equipped with a detector of ELSD 3300/2000 and a TSK-gel G5000 column (7.8 mm × 30 cm, Tosoh (Shanghai)Co. Ltd., Shanghai, China). Standard T-series dextrans with molecular weights of 10, 40, 70, 150, 220, 300, 380 kDa were used at 1% (w/v) concentration. The standard solution of the T-series and glucose (10 μL) were passed through the column using isocratic mode at a flow rate of 0.8 mL/min with deionized water as a mobile phase at a column temperature of 30 °C, the elution times were plotted against the logarithm of their respective molecular weights.

#### 3.3.6. Fourier Transform Infra-Red Spectroscopy (FTIR) of PBLPS

PBLPS-1 was directly measured by FTIR spectroscopy in a range of 4000 to 400 cm^−1^ using KBr disks containing 1% finely ground samples on a Nicolet 550 FT-IR spectrometer (Thermo Nicolet, Waltham, MA, USA). FTIR is used to investigate the vibrations of molecules and polar bonds between the different atoms, types of monosaccharide, glycosidic bonds and functional groups [20].

#### 3.3.7. NaIO_4_—HIO_4_ Oxidation and Smith Reduction of PBLPS

Standard solutions of 0.15 mol/L sodium periodate and sodium iodide (50 mL) were prepared. Sodium periodate solution and sodium iodide solution were mixed in proportions of 5:0, 4:1, 3:2, 2:3, 1:4, and 0:5 (v/v), respectively, and a sample of the mixture (0.4 mL) was placed in a 100 mL volumetric flask, deionized water added to dilute the solution to the mark, then the standard curve of sodium periodate and sodium iodide solution was produced by the UV absorbance value measured at 223 nm.

PBLPS sample (30 mg) was placed in 250 mL brown flask, and 0.015 mol/L solution of sodium periodate (60 mL) was added into the flask with shaking to dissolve the sample, then placed in the dark at 4 °C with intermittent oscillation. Aliquots (0.2 mL) of reaction solution were placed in a 50 mL volumetric flask at predetermined time intervals for UV analysis, and the rest was used for Smith hydrolysis. The amount of formic acid generated in the reaction was determined by titrating 5.0 mL of the reaction solution with 0.0086 mol/L sodium hydroxide, adding 2 drops of ethylene glycol, phenolphthalein as indicator for 30 min. According to the method [21], the products of periodate oxidation was dialyzed for 48 h, and concentrated under reduced pressure below 40 °C to about 10 mL of total volume, 1 mol/L H_2_SO_4_ was added (2 mL) and completely hydrolyzed at 100 °C for 8 h. The hydrolysate was neutralized with BaCO_3_, then filtered. The filtrate was concentrated and determined by GC analysis after acetylation.

## 4. Conclusions 

The total polysaccharides of 32 samples of bamboo leaves was between 1.4% and 5.4%, while BLPSC of PN, PV, CQ and PB was 4.2%–5.4%, which was about 2–3 fold more than in samples No. 1–No. 28 of the different species. Particularly the content of galactose for the four typical bamboo leaves was in a range of 21.5%–34.1%, which is also definetly 2–3 fold more than in No. 1–No. 28. Based on the results of sugars analysis, molecular weight determination, FTIR spectra, NaIO_4_—HIO_4_ oxidation and Smith degradation as well as the existing literature concerining the structural properties of bamboo polysaccharides, all PBLPS-1 are homogeneous polysaccharides with molecular weights of 10^4^–10^5^ Da, while PBSP-2 are heterogeneous polysaccharides with two or three kinds of polysaccharides. PB-PBLPS-1 is a mixture of α-galactopyranose linkages and β-d-glucopyranose linkages with α-(1→6) or β-(1→6) glycosidic bonds, while PN-PBLPS-1, PV-PBLPS-1,and CQ-PBLPS-1 are just α-galactopyranose linkages of α-(1→6)glycosidic bonds.

In general, hemicelluoses or polysaccharides from bamboo wood or bamboo shoot consist of d-xylose, l-arabinose, d-galactose, d-mannose, d-glucuronic acid, 4-*O*-methyl-d-glucuronic acid, d-galacturonic acid and lesser amounts of l-rhamnose, l-fucose, and various *O*-methylated neutral sugars, which contain a backbone of d-xylopyranosyl residues, linked together by β-(1→4)-glycosidic bonds. These hemicellucosic polysaccharides have an average Mr range of over 10^5^ Da and very weak physiological activity. However, the polysaccharides of bamboo leaves (BLPS) is a kind of bioactive heteropolysaccharide with relative medium molecular mass of less than 10^4^ Da. BLPS had been proved to have good antitumor physiological activity and has been used widely as edible and medicinal materials as well as cosmetic additives. This study found high content and bioactive polysaccharides in the four varieties *P. nigra* (Lodd.) Munro (PN), *P. vivax* McClure (PV), *Chimonobambusa quadrangularis* (Fenzi) Makino (CQ), and *P. bambussoides* cv. tanakae (PB). These bioactive polysaccharides are mainly composed of xylose, galactose, and glucose, furthermore, the structural characterization of mono-sugar link styles were preliminary elucidated as glucopyranose linkages with glycosidic bonds, therefore, this work will provide a basis to research the fine structure and biological activity of polysaccharides from these four typical kinds of bamboo leaves.
